# Poverty and health among CDC plantation labourers in Cameroon: Perceptions, challenges and coping strategies

**DOI:** 10.1371/journal.pntd.0006100

**Published:** 2017-11-20

**Authors:** Valerie Makoge, Lenneke Vaandrager, Harro Maat, Maria Koelen

**Affiliations:** 1 Health and Society (HSO) group, Wageningen University and Research, Wageningen, The Netherlands; 2 Institute for Medical Research and Medicinal Plant studies (IMPM), Yaoundé, Cameroon; 3 Knowledge Technology and Innovation (KTI) group, Hollandseweg 1, Wageningen University and Research, Wageningen, The Netherlands; Walter and Eliza Hall Institute, AUSTRALIA

## Abstract

Creating better access to good quality healthcare for the poor is a major challenge to development. In this study, we examined inter-linkages between poverty and disease, referred to as poverty-related diseases (PRDs), by investigating how Cameroon Development Corporation (CDC) camp dwellers respond to diseases that adversely affect their health and wellbeing. Living in plantation camps is associated with poverty, overcrowding, poor sanitation and the rapid spread of diseases. In a survey of 237 CDC camp dwellers in Cameroon, we used the health belief model to understand the drivers (perceived threats, benefits and cues for treatment seeking) of reported responses. Using logistic regression analysis, we looked for trends in people’s response to malaria. We calculated the odds ratio of factors shown to have an influence on people’s health, such as food, water, sanitation challenges and seeking formal healthcare for malaria. Malaria (40.3%), cholera (20.8%) and diarrhoea (17.7%) were the major PRDs perceived by camp dwellers. We found a strong link between what respondents perceived as PRDS and hygiene conditions. Poverty for our respondents was more about living in poor hygiene conditions than lack of money. Respondents perceived health challenges as stemming from their immediate living environment. Moreover, people employed self-medication and other informal health practices to seek healthcare. Interestingly, even though respondents reported using formal healthcare services as a general response to illness (84%), almost 90% stated that, in the case of malaria, they would use informal healthcare services. Our study recommends that efforts to curb the devastating effects of PRDs should have a strong focus on perceptions (i.e. include diseases that people living in conditions of poverty perceive as PRDs) and on hygiene practices, emphasising how they can be improved. By providing insights into the inter-linkages between poverty and disease, our study offers relevant guidance for potentially successful health promotion interventions.

## Author summary

Poverty is a condition that increases disease risks and presents severe health challenges. The negative impact of poverty on health is well understood, but much less is known about how people living in poor conditions themselves perceive health challenges and how they try to overcome these challenges. We studied a group of Cameroon Development Corporation (CDC) plantation workers, living in housing provided by the company, known as camps. Living conditions in plantation camps are characterised by small and very basic housing, shared toilet facilities and overall poor sanitation. In the camps, water sources were public taps found in strategic areas. During water cuts, camp dwellers used streams as their water sources. Water was treated at CDC management level. We questioned 237 labourers (or dependants) on matters related to their health. Malaria, cholera and diarrhoea were reported as the main diseases that they associated with poverty. Our results also show that the labourers associated poverty primarily with the poor state of their living conditions rather than with not having money. Even though CDC offered free healthcare, camp dwellers relied on self-treatment and unofficial medication, especially for malaria for which almost 90% of the workers did not use the free services. Therefore, for interventions to be successful, they should include people’s perceptions and focus on improving hygiene conditions.

## Introduction

Creating better access to good quality healthcare for the poor is a major challenge to development [1]. Poverty is a condition that increases disease incidence for many people in developing countries [2]. The World Health Organisation (WHO) reports that up to 45% of disease cases in developing countries result from poverty [3]. The perceived relation between poverty and disease has resulted in the term poverty-related diseases (PRDs), which illustrates that poverty is a reason for disease presence and a limiting factor in its effective management and cure [4]. Socio-economic status (SES) has been shown to influence the burden of diseases in low and middle income countries [5–7]. However, behind the daunting statistics on PRDs lies a complex reality. In most situations, poverty implies that disease prevention and treatment are available erratically or partially, and this increases the challenge to effectively manage diseases [4, 8]. Another challenge to the effective management of diseases, especially in the case of interventions, relates to people’s perceptions of diseases. These perceptions influence the ways in which people respond to diseases [7, 9, 10]. Understanding perceptions is important for creating meaningful programmes and messages for successful interventions [11]. Understanding the role of perceptions in disease management, especially in people living in conditions of poverty, throws more light on some of the inter-linkages between poverty and health. A deeper understanding of these inter-linkages is important for the proper design, implementation and evaluation of health promotion interventions, especially those aimed at PRDs.

The top three major PRDs reported by international organisations such as the WHO are malaria, HIV/AIDS and tuberculosis (TB) [3]. These diseases together are responsible for 18% of the disease burden in the poorest countries [8]. The persistence of PRDs leads to a reduced quality of life and wellbeing for people and hinders economic growth in developing countries [12].

In Cameroon, diseases listed as PRDs are very common. For instance, malaria accounts for most hospitalisations and absences from work [13]. HIV accounts for about 114 new infections daily, with the highest prevalence rate recorded in the South West Region of Cameroon [14]. The presence of HIV has led to an increase in the number of TB cases in the country, making it the third listed major PRD [15, 16]. People may avail of healthcare services to respond to PRDs, but they usually have to pay out of their own pocket, with a consequent heavy toll on family budgets [17, 18].

In order to get a better understanding of the inter-linkages between poverty and diseases, we designed a study of people’s responses to health challenges in two distinct groups of people in Cameroon. These groups were students at two state universities [19] and plantation workers (and dependants of workers) of the Cameroon Development Corporation (CDC). In this paper, we focus on the survey carried out in CDC camps. Camps are settlements in which plantation workers live. Living in plantation camps has been associated with overcrowding, poor sanitation and the rapid spread of diseases [20]. Despite having a paid job, people in plantation camps face financial challenges because their salaries are very low. Such conditions have significant consequences for the kinds of decisions or actions in which they engage with respect to their health [21–23]. The aim of this study was to acquire a deeper understanding of the inter-linkages between poverty and disease by investigating how CDC camp dwellers respond to diseases that adversely affect their health and wellbeing. In order to achieve this aim, we drew on stipulations of the health belief model [24,25].

**Fig 1:**
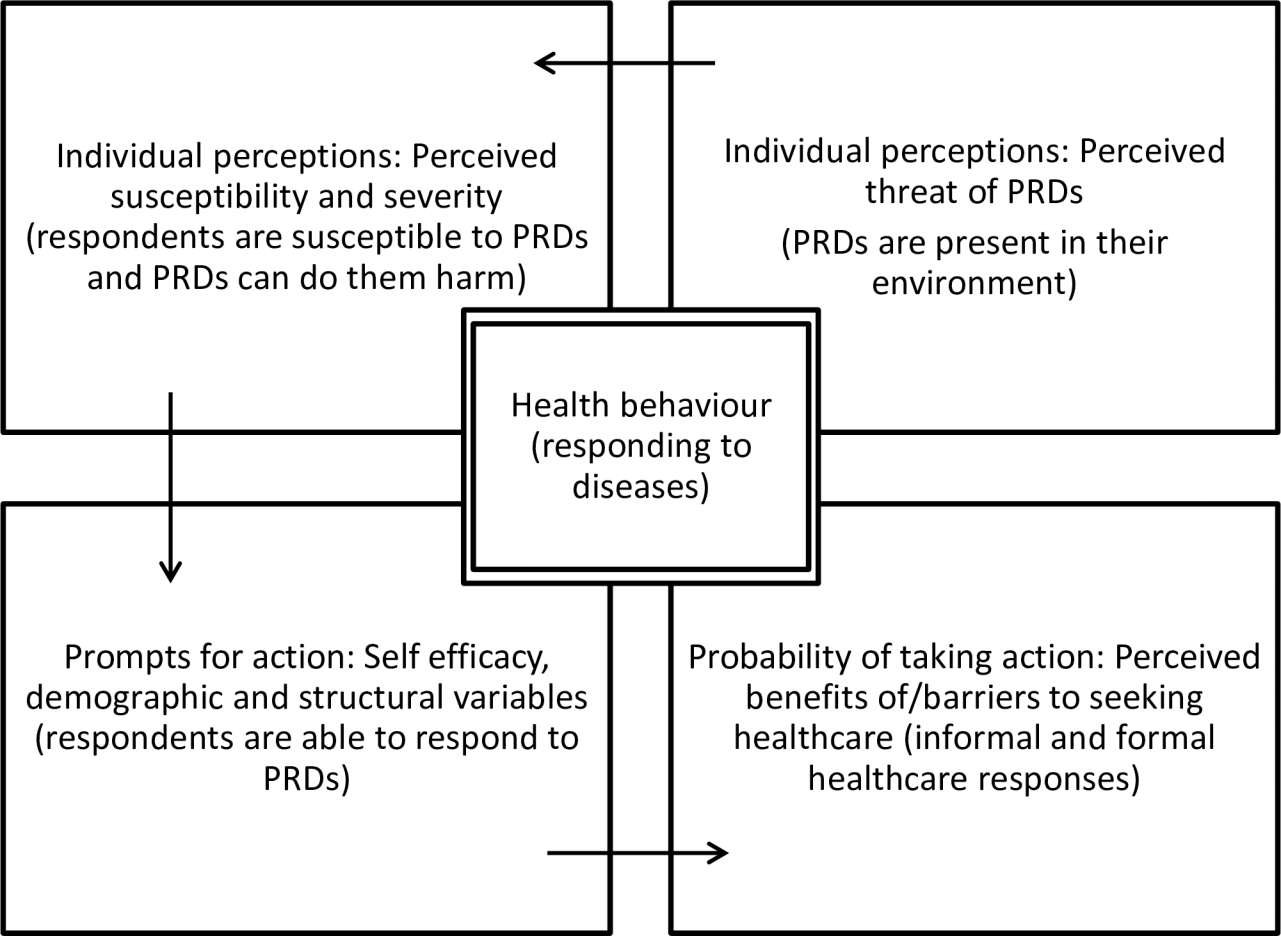
Operationalisation of the health belief model around here.

The health belief model (HBM) (see operationalisation in Fig 1) indicates that a health behaviour is determined by two main beliefs. The first is a belief in the presence of a health threat, characterised by perceived susceptibility towards disease as well as perceived severity of the disease. This implies, in our case, camp dwellers’ perception of the risk of being infected by PRDs and the distress this can cause. The second is a belief in the perceived benefits of, and perceived barriers to, engaging in a health behaviour. These perceptions constitute a belief about the effectiveness of the actions taken to remedy the situation [25–27]. The HBM offers a tool with which we can obtain a deeper understanding of how and why people living in CDC camps in Cameroon respond to diseases in the ways they do. Proper understanding of disease responses by CDC camp dwellers is essential for the development of successful efforts to promote health in plantation camps.

## Methods

### Ethical statement

Ethical approval for this study was provided by the Wageningen University review board and the health and human resource departments of CDC. The aim of the study was carefully explained to all respondents. Respondents signed an informed consent form before participating in the study.

### Survey design and respondents

Our study was part of a larger project seeking to unravel complexities between poverty and health/diseases, focusing on disease responses and coping strategies among respondents from two distinct settlements: CDC camps and university campuses in Cameroon. Fieldwork for the larger study took place between February 2013 and July 2014. The results obtained from the study involving the students have been published elsewhere [19]. The study design of the research presented here is a survey.

CDC is an agro-industrial organisation, located in the South West Region of Cameroon, formed in 1947 with the aim of developing tropical crop plantations for export and the national market. It runs banana, rubber and oil palm plantations. As a corporation, CDC offers its workers free healthcare services and free housing in settlements, called camps, usually found close to the plantations it runs. CDC has two types of camps: those close to a big town and others further away from big towns. Camps close to big towns are more populated than those further away. Most CDC labourers have low education level. Camp respondents who participated in this study had jobs as tappers, harvesters, camp and compound workers, weeders, security guards, toilet cleaners, clearers and dependants.

The camps selected for our study included one camp close to a major town (Limbe), called Limbe camp, 4.0225° N, 9.1954° E and two other camps, remote from a major town, namely, Sonne camp and Camp 7, 4.0786° N, 9.3590° E. These three camps vary in size. Sonne and Camp 7 each house about 50 households, whereas Limbe camp has more than 200 hundred households. The houses in the camps are organised in rows. These houses are mostly in a run-down condition. Congestion characterises life in the camps because most families have only two rooms allocated to their families. In such cases, kitchens are transformed into bedrooms, or zinc or wood extensions are added to houses as extra room space. Unreliable water and electricity supply is common in the camps. People living in the different CDC camps face similar problems with respect to living conditions, even though some camps are in a worse state than others in terms of having basic necessities like a reliable supply of water and electricity.

In order to be eligible for this study, respondents had to be living in a CDC camp as a worker or a worker’s dependant. In Limbe camp, sampling of respondents followed what has been previously described by Moyou et al [29] whereby, for a population of less than 1,000 inhabitants, one out of every two houses is selected for the research. In Sonne camp and Camp 7 however, all houses were visited and a member was requested to participate because these camps were relatively small and had relatively fewer inhabitants. We envisaged getting one member (not necessarily the household head as we wanted a broad view on ways of experiencing PRDs and management strategies) to fill out the questionnaire per household visited. The camps were visited during hours when workers had returned from the field/farm, and this was usually after midday.

### Survey instrument

The survey instrument used for this study was as previously described in our study of university students in Cameroon [11]. From initial observations and preliminary conversations with students and camp dwellers, we obtained an image of the key issues and conditions affecting students’ and camp dwellers’ health, from which we designed a questionnaire in line with the HBM [24]. The questionnaire was comprised of closed questions with yes/no answer options and questions with pre-defined answer categories, with the possibility for multiple responses in some questions.

### Respondents’ individual perceptions

Perceived threat in our study was operationalised as a perceived susceptibility to diseases [24,30]. Respondents were asked what diseases they perceived as common and what diseases they associated with poverty. Options for PRD choices, namely, cholera, diarrhoea, typhoid fever and meningitis were included in our study in addition to those listed as the top three by health agencies such as the WHO. Questions further centred on previously reported aspects with a direct impact on health, namely, food availability, balanced diet, good and permanent water, and sanitation challenges [31,32]. Perceived severity of disease was not investigated because this aspect has already been reported in other studies [10,14,33,34].

### Respondents’ prompts to action

Prompts to action can be understood as the camp dwellers’ perception that they can take actions to respond to diseases that they face. This was based on their believing that they were able either to carry out actions needed to manage prospective situations or to take preventive actions to avoid PRDs or perceived threats [35,36]. A question about response to malaria was included because malaria is commonplace in the country [34,37].

### Probability of respondents taking action

Perceived benefits of, and barriers to, the effectiveness of a contemplated action would turn prompts to action into actual action [24]. Several possibilities for actions in the formal and informal medical sectors were included in the questionnaire. Finally, also included were questions relating to socio-demographic variables (age, sex, education, marital status and income) because of their role in people’s responses to disease [28].

### Procedure

Prior to the actual survey, draft questionnaires were pre-tested amongst labourers at the CDC head office to ensure that the questions were comprehensible. Following from this pre-test, a few questions were reformulated for greater clarity.

All questionnaires were in English. The questionnaires were either self-administered or administered interview-style depending on whether the respondent was literate or capable of self-administering the questionnaire. In the interview-style administration, questions were read out to respondents in English and translated into Pidgin English when preferred by the respondent.

The first household selected for the distribution of questionnaires was the one closest to the entrance to the camp. In the case of Limbe camp, we then moved on to other houses in the camps, selecting one out of every two houses and requesting participation. The questionnaires were left with those who could self-administer it and picked up later in the day. In Camp 7 and Sonne camp, where all households were asked to participate, questions were asked interview-style, and the first author or a trained assistant noted down responses given. A total of 237 camp dwellers completed the questionnaire out of a possible 247, giving a response rate of 95.9%.

### Data analysis

The data were analysed using SPSS version 22 (SPSS Inc. IBM). Before analysis, data from pre-coded questionnaires were entered into SPSS and checked for errors by the first author and an assistant. Chi-square tests were used to analyse differences between the camps as well as other factors included in the survey, such as general disease response strategies and malaria response strategies as well as using formal healthcare services. We performed logistic regression analysis to explain patterns observed in the way people responded to malaria. Socio-demographic factors (age, sex, educational status, marital status and income) served as predictor variables. The odds ratio of factors shown to have an influence on people’s health, such as food, water and sanitation challenges, and seeking formal healthcare for malaria, was calculated. Paired sample t-tests were carried out in order to compare mean difference between two sets of observations namely, the observation of respondents’ specific response to malaria and the observation of the same respondents’ response to diseases in general; *p* values <0.05 were considered to be significant.

## Results

### Respondents’ characteristics

The demographic distribution of the respondents is shown in Table 1. Of the 237 camp dwellers that participated in this study, 56.4% were male and 43.6% were female. Half of the respondents were married. The income of most camp dwellers was between 20,000 FCFA and 50,000 FCFA (between 33 and 82 US$) per month. Respondents from Sonne camp and Camp 7 had basic primary school education or no formal education. About 40% of respondents from Limbe camp had received education at secondary school level. Differences were seen among the different camps in relation to age, education and income. In terms of numbers, 160 respondents were from Limbe camp. This is one of the biggest camps owned by CDC. Limbe camp had more respondents who were younger, more educated and unmarried than Sonne camp and Camp 7 (Table 1).

**Table 1:** Background characteristics of CDC camp respondents.

		Camp settings			
		Limbe camp (n = 160)	Camp 7 (n = 43)	Sonne camp (n = 34)	*p* value*
		% (n)	% (n)	% (n)	
Sex	male	53.5 (85)	55.8 (24)	70.6 (24)	0.188
	female	46.5 (74)	44.2 (19)	29.4 (10)	
Education	no formal education/FSLC	59.7 (80)	97.6 (40)	97.1 (33)	0.000
	secondary education	40.3 (54)	2.4 (1)	2.9 (1)	
Participants’ age in ranges ϯ	< 25	17.7 (28)	9.3 (4)	0.0 (0)	0.001
	25─34	34.2 (54)	32.6 (14)	11.8 (4)	
	35─44	29.1 (46)	39.5 (17)	38.2 (13)	
	45─54	13.9 (22)	14.0 (6)	38.2 (13)	
	55 and over	5.1 (8)	4.7 (2)	11.8 (4)	
Marital status	married	39.0 (60)	69.8 (30)	79.4 (27)	0.000
	single	61.0 (94)	30.2 (13)	20.6 (7)	
Participants’ income level	<20thousandFCFA	25.2 (40)	26.2 (11)	0.0 (0)	0.011
	20─50thousand FCFA	52.2 (83)	64.3 (27)	79.4 (27)	
	50─100thousandFCFA	15.7 (25)	4.8 (2)	17.6 (6)	
	>100thousandFCFA	6.9 (11)	4.8 (2)	2.9 (1)	

Ϯ Youngest participant was aged 16 years

*p-values are from chi square analysis

Chi square analysis did not reveal a significant effect of any of the background variables on the way respondents responded to diseases. Therefore, we considered the three camps as a single group of CDC camp respondents.

### Respondents’ belief in diseases as a threat and perceived vulnerability towards PRDs

The top three diseases identified by respondents as most common were malaria: 93.2%, typhoid: 27.7% and diarrhoea: 15.7%. However, answers given to the question on what respondents perceived as major PRDs showed malaria, cholera and diarrhoea as the top three. Major PRDs listed by WHO, namely, HIV/AIDS and TB, were not highly rated by our respondents (Table 2).

**Table 2:** Respondents’ classification of common diseases and PRDs (camps).

Diseases	*Diseases % (n)	
	Common diseases	PRDs
Malaria	**93.2 (219)**	**40.3 (91)**
Typhoid fever	**27.7 (65)**	8.8 (20)
Diarrhoea	**15.7 (37)**	**17.7 (40)**
Cholera	10.2 (24)	**20.8 (47)**
HIV/AIDS	7.2 (17)	14.2 (32)
TB	2.6 (6)	4.4 (10)
STIs	1.7(4)	3.5 (3)
Meningitis	0.9 (2)	na

*More than one response was possible; na = not asked

The main cause attributed to disease presence was (lack of) hygiene (72.3%). In this study, up to 54% of respondents reported that they shared toilet facilities with more than 10 other households. Other causes attributed to disease presence were lack of knowledge (23.4%), poverty (17.9%), poor education (14%) and climate (14%).

### Prompts for action: Respondents’ response to diseases

The different ways in which camp dwellers respond to diseases based on their socio-demographic characteristics are indicated in Table 3. Using formal healthcare services was reported as the way in which most respondents would generally respond to diseases. This was independent of socio-demographic characteristics.

**Table 3:** Variation in health-seeking practices by socio-demographic differences.

		Formal	Informal	Both	*p* value
		(n) %	(n) %	(n) %	
Sex	male	(108)83.7	(18)14.0	(3)2.3	0.768
	female	(81)84.4	(14)14.6	(2)1.0	
Education	no formal education/FSLC	(118)80.8	(26)17.8	(1)1.4	0.476
	O Level/A Level	(47)90.4	(4)7.7	(1)1.9	
Participants’ age in rangesϯ	< 25	(22)78.6	(6)21.4	(0)0.0	0.720
	25─34	(62)89.9	(6)8.7	(1)1.4	
	35─44	(63)85.1	(9)12.2	(2)2.7	
	45─54	(29)74.4	(9)23.1	(1)2.6	
	55 and over	(12)85.7	(2)14.3	(0)0.0	
Marital status	married	(92)81.4	(20)17.7	(1).9	0.110
	single	(92)86.0	(12)11.2	(3)2.8	
Employment status	employed for wages	(137)85.6	(20)12.5	(3)1.9	0.153
	housewife	(16)88.9	(2)11.1	(0)0.0	
	student	(22)75.9	(7)24.1	(0)0.0	
	self-employed	(13)76.5	(3)17.6	(1)5.9	
	retired	(2)100	(0)0.0	(0)0.0	
Participants’ income level in ranges	<20thousandFCFA	38)82.6	(7)15.2	(1)2.2	0.161
	20─50thousandFCFA	(110)83.3	(20)15.2	(2)1.5	
	50─100thousandFCFA	(31)96.9	(1)3.1	(0)0.0	
	>100thousandFCFA	(10)71.4	(3)21.4	(1)7.1	

Ϯ Youngest participant was aged 16 years. Note: Figures may not add up to exactly 100% because of rounded values.

### Respondents’ response to malaria

The reported use of formal healthcare services to respond to diseases in general (84.1%) changes remarkably when it comes to malaria. Results from a paired samples t-test showed a significant difference (t (225) = 23.520, *p* = .000). These inconsistencies are illustrated in Table 4. Regarding malaria, the use of informal treatment and medication was indicated as the preferred response (88.5%), with only about 5% of respondents reporting the use of formal healthcare services.

**Table 4:** Inconsistencies in disease responses towards malaria and other diseases.

	Formal	Informal	Both	*p* value
Health-seeking practices in general	84.1%	14.2%	1.8%	0.000
Health-seeking practices in the case of malaria	4.7%	88.5%	6.8%	

In order to explain observed inconsistencies, we sought to find out which socio-demographic factors would predict seeking formal healthcare in the case of malaria. Results from logistic regression analysis, presented in Table 5, showed that socio-demographic factors were not predictors of the healthcare choices in the case of malaria.

**Table 5:** Logistic regression model with seeking formal healthcare in the event of malaria as dependent variable.

Variables	B	S.E.	Sig.	Exp(B)
Age	.025	.035	.482	1.025
Sex	-.024	.641	.971	.977
Educational status	-.662	.901	.462	.516
Marital status	1.173	.737	.111	3.233
Income	.205	.448	.646	1.228
Nagelkerke R2				.045

Information regarding the presence or absence of water, sanitation and food challenges faced by respondents and the choice of a formal, an informal or both types of response to malaria can be seen in Table 6. Responding to malaria using the informal sector did not vary whether or not respondents faced sanitation, food and water challenges. Also in Table 6 is shown the odds ratio analysis of people who were facing these challenges and using formal healthcare services as a response to malaria. The odds ratio indicated that only respondents faced with the challenge of unreliable water facilities showed higher odds of using formal healthcare services for malaria. The other challenges did not show significant results.

**Table 6:** Response to malaria in the presence of sanitation, food and water challenges and odds ratios at 95% CI for seeking formal healthcare in the presence of these challenges.

Sanitation, food, and water challenges (n)		Response to malaria					
		Formal %	Informal %	Both %	Odds ratio		P value based on odds ratio
Toilet sharing with other houses	Yes (224)	4.7	84.7	6.0	0.497 (.062–4.004)		0.503
	No (11)	-	3.8	0.9			
Water cuts in the neighbourhood	Yes (117)	3.8	43.6	2.6	0.261 (.117-.583)		0.001
	No (117)	0.9	44.9	4.3			
Food readily available for the participant	Yes (78)	-	27.9	5.6	1.727 (.772–3.861)		0.179
	No/sometimes (155)	4.3	60.9	1.3			
Participant cooks his/her meals	Yes/sometimes (176)	3.8	65.5	5.5	0.894 (.396–2.017)		0.787
	No (59)	0.9	23.0	1.3			
Participant considers his/her diet to be balanced	Yes (151)	3.0	55.3	6.0	0.690 (.323–1.475)		0.337
	No (84)	1.7	33.2	0.9			
Participant misses one or more meals	Yes/sometimes (183)	3.8	71.9	2.1	0.909 (.389–2.125)		0.826
	No (52)	0.9	16.6	4.7			

Note: Values on table may not add up to exactly 100% because of rounded values.

### Prominent informal healthcare responses to malaria

Almost 80% of respondents reported using small pharmacies as their primary means of malaria treatment. A small pharmacy refers to any small shop selling medication. These outlets are different from big pharmacies in the sense that they are not registered with the government as the big ones are. Another way of dealing with malaria was by carrying out self-medication practices, reported by 13.5% of respondents. In self-medication, a combination of different medicinal plants is brewed as tea and consumed to prevent and treat malaria. Common medicinal plants reportedly used were mango leaves, pawpaw leaves, guava leaves, aloe-vera and fever grass.

### Probability of taking action: Determinants for seeking formal and informal healthcare

On the question of how soon respondents would seek formal healthcare when ill, 51.9% of respondents reportedly sought formal healthcare at the onset of disease symptoms, 20.4% when illness was severe and 20% only after self-medication had failed.

Factors that determined the use (or not) of formal healthcare services are presented in Table 7. Having money (reported by 50.6%) was mentioned most often as a factor enabling people to avail of official medical facilities (other than the free-of-charge CDC facilities), followed by unavailability of drugs at home (22.1%), duration of illness (12.6%), severity of illness (12.6%) and attitude of hospital staff (10.4%). Analysis of the association between reported use of formal facilities and the determinants showed that only severity of illness (*p* = 0.004) and time (*p* = 0.023) are significantly associated.

**Table 7:** Determinants for use of formal healthcare facilities.

Determinants		Using Formal healthcare services		
	% (N)*	Formal response (Yes)	Formal response (No)	*p* value
Money	50.6 (115)	100	15	0.199
Unavailability of drugs at home	22.1 (51)	46	5	0.162
Duration of illness	12.6 (29)	26	3	0.363
Severity of illness	12.6 (29)	19	10	0.004
Attitude of hospital staff	10.4 (24)	18	6	0.213
Distance to healthcare service	7.8 (18)	14	4	0.466
Time	0.4 (1)	1	0	0.023
Fear	0.4 (1)	0	1	0.660

* More than one response was possible

## Discussion

The aim of this study was to acquire a deeper understanding of the inter-linkages between poverty and disease by investigating how CDC camp dwellers in Cameroon respond to diseases that adversely affect their health and wellbeing. Such investigations provide insights needed to properly design interventions to improve people’s health conditions.

Our study showed that camp dwellers differentiated between what they perceived as common diseases and what they perceived as PRDs. Malaria, for example, is considered as both a common disease and a PRD. Typhoid fever is considered a common disease but is not named among major PRDs, whereas cholera is perceived as a major PRD but not as a very common disease (see Table 2). These perceptions of PRDs differ from the top three in official lists (malaria, HIV and TB) issued by international and national health agencies such as the WHO or the Cameroon ministry of public health (MINSANTE) and highlight complexities that exist in respondents’ disease classification [3,33]. Respondents’ perceptions vis-à-vis diseases must be considered in any intervention because perceptions have been shown to influence the way people respond to diseases [7, 9, 10, 38]. Our study revealed that diseases associated with poor hygiene conditions are those that are perceived by respondents as being poverty-related. The exact reason for the difference in PRD classifications by respondents and by official reports is unclear, but if the main reported attributed cause for disease presence, i.e. poor hygiene conditions, is considered, then the perceived strong link between common diseases, PRDs and poor hygiene indicates a reason for camp dwellers’ classification. By implication, poverty for our respondents is more about living in poor hygiene conditions than lack of money. Health challenges are not about lack of access to healthcare services, as workers and their dependants have free access to CDC clinics, but rather about coping with the consequences caused by the immediate living environment. Even though access to healthcare services did not pose health challenges for camp dwellers, previous research showed that the services received at these clinics entailed other challenges in terms of the quality of medication, the attitude of clinic staff and the constant unavailability of medication [20,39]. In the camps, therefore, the immediate living environment acts as an element that exacerbates poor hygiene conditions and the spread of diseases [40].

Efforts to curb the devastating effects of PRDs, then, should focus strongly on hygiene practices and how they can be improved. The report of families sharing toilet facilities with more than 10 other households in the camps underlines this point. Such conditions increase the risk of infections and the spread of diseases and are potentially fertile ground for the spread of epidemics [41]. Respondents mentioned poverty and climatic conditions as reasons for common disease presence. Such attributions did not rate as highly as poor hygiene conditions. This once again highlights where the focus should lie in designing health promotion interventions.

The way people perceive diseases, the attributions they indicate for disease presence and the perceived effectiveness of health actions against diseases influence how people respond to these diseases [25]. The HBM enables a deeper understanding of how these different factors interact and lead to the final decision on which course of action to take [24]. Our study revealed that responses to diseases varied. Generally, the dominant response is to seek formal healthcare (84.1%). This is understandable, as camp dwellers have the option of free healthcare services offered by CDC, which seeks to promote the health and wellbeing of its workers [42]. However, it is interesting to see that, in the case of malaria, the most common PRD, almost 90% of respondents reported that they would use informal healthcare practices. Similar results were found in our study among students [19].

One of the reasons for this inconsistency could be that malaria is very common [34,43]. Although malaria is very common, headache and fever, the early symptoms of malaria, are also very common even though not particular to malaria. Crump et al [44] in their study on malaria diagnosis pointed out that the error rate for malaria can be quite high. This may imply that in many instances taking informal medication on the basis of early symptoms is effective in making the symptoms go away, probably because they are not caused by malaria. This sustains the belief that informal medication is effective against malaria and maintains the practice. Camp dwellers’ experience in dealing with malaria in ways other than hospital-based care reveals the complexities linked to malaria and an overall inefficiency of official healthcare facilities due to the lack of an integrated approach to fighting malaria. The HBM helps to explain this, as responding to a disease with the intention of being cured is determined by a belief in a health threat and a belief in the effectiveness of the response chosen, e.g. formal or informal practices sought for treatment and cure. The use of small pharmacies and self-medication practices are therefore perceived to be equally beneficial, if not better, for getting cured. Moreover, the results from our qualitative study in the same setting [36] revealed that people disliked drugs prescribed at the CDC healthcare services for malaria because of their severe side effects. Respondents reported that this was a factor keeping them away from the clinics [39]. Our results reveal that an offer of free medical care is not sufficient to cause people to use the facilities. This is contrary to reports in another study, which indicated that lack of financial means prevented people from using formal healthcare services [45]. In the camps, other factors, for example treatment quality, also matter when services are free as explained above. Such factors are perhaps more important than financial concerns, as confirmed in other studies. In Ghana for example, even though the government abolished fees for hospital-based care for expectant mothers, these women did not avail of this because of a lack of trust in the system [46]. CDC would probably see an increase in the use of the services it offers if it revisited its current treatment policy against diseases such as malaria. Providing more effective drugs in a consistent manner and improving workers’ living conditions would go a long way towards establishing an integrated approach to fighting malaria.

It has been reported previously that poor socio-demographic status plays a role in people’s utilisation of the formal healthcare system [47]. This was not the case in our study, as none of the socio-demographic factors predicted using formal healthcare in the case of malaria. That notwithstanding, when threats to people’s health and wellbeing such as the lack of basic necessities (permanent water supply, toilet facilities, affordable and available food) are considered, our study showed that only people who experienced water cuts had higher odds of seeking formal healthcare in the case of malaria. The reason for this is not very clear. Unreliable and poor water supply is linked to an increase in unsanitary conditions [48] and increased risk of infectious diseases such as malaria. An increased frequency of infections, therefore, could be a reason why these people seek the free formal healthcare services offered by CDC. Further research is needed to clarify this.

Respondents reported factors that would facilitate their use of formal healthcare facilities to respond to diseases. Having money was the most important reason enabling formal response to disease. This confirms what has been reported in other studies [49,50]. In the CDC case, as workers and their dependants can receive free treatment at the hospital, having money would probably be an incentive to seek other ‘better’ formal healthcare facilities that do not belong to CDC. Seeking such treatment would therefore require out-of-pocket payment. Other triggers were unavailability of drugs at home, duration of illness and perceived severity of diseases. Our results add to studies on determinants of utilisation of formal healthcare services in countries and confirms perceived severity of disease as an incentive for hospital-based care [1, 2, 50, 51].

Our study has revealed the way people in CDC camps respond to diseases that adversely affect their health and wellbeing. It provides insights that help to explain why a disease like malaria is such a problem in Cameroon. In a parallel study on university students in Cameroon [19], we showed that students, with a much better educational background than the CDC workers, respond to malaria in very similar ways. Because students have to pay for official healthcare, reliance on self-medication practices and informal medication was even more prominent, especially for malaria. It is interesting to note that such different groups of people have the same responses towards malaria. As malaria is still a major public health problem [43], our results could mean that informal responses to malaria probably contribute to its persistence as a public health problem. Informal responses cannot be eliminated. By using the HBM, we have been able to highlight the repertoire of options people have available to respond to malaria (self-medication and small pharmacies). We therefore recommend that government policy and also CDC policy should include seeking to improve informal practices such as self-medication for malaria and also aim to train informal healthcare providers such as vendors in small pharmacies so that they will be able to administer the right medication in the right dosages.

### Study limitations

Our respondents were from CDC camp settings, which is a particular group of respondents. By focusing primarily on this group, our study may be limited in its external validity across other non-camp settings. Also, we used the HBM in this study. A limitation with this model is that there may be reasons (e.g. social) why people engage in health behaviour other than trying to avoid a negative health outcome, and these would be ignored by the model. That notwithstanding, use of the HBM elucidated inter-linkages between poverty and health in a poor setting, and this is essential for the design of health promotion interventions in poor countries.

## Conclusion

Our study showed that camp dwellers perceived differences between common diseases and PRDs and attributed the latter to poor hygiene conditions characteristic of their dwelling place. Our study also revealed a list of major PRDs (malaria, cholera and diarrhoea) different from those listed by international and national health agencies. The classification brought forward by our respondents is based on what they perceive to be health threats. Our study has shown that such perceptions are formed on a composite understanding of health, including factors that exceed the strict physiological and biological principles of medical disease definitions. We argue that understanding people’s perceptions is important for health agencies and medical service providers addressing PRDs because it creates opportunities to support people in responding to diseases more effectively. This also indicates a need to increase the range of major PRDs from malaria, HIV and TB to include cholera and diarrhoea. Our respondents’ association of poverty and related diseases with the conditions of the immediate environment highlights the fact that poverty has to be understood as being more than lack of money or not having access to healthcare services. Camp dwellers showed that they played an active role in matters concerning their health and wellbeing. This is highlighted by the fact that, despite free healthcare services offered by CDC, almost 90% of respondents did not immediately use these services for malaria treatment. They instead engaged in informal treatment practices including the use of small pharmacies and self-medication practices. By so doing, they showed that resolving health challenges is not limited to using free health services but extends to employing a repertoire of options that are available to them.

It is the duty of the Cameroon government and other organisations in Cameroon such as the CDC to seek to reduce the burden posed by PRDs. Prompt and effective diagnosis, management and treatment of PRDs is essential but not sufficient to control PRDs. A greater understanding of the inter-linkages between poverty and health or poverty and disease, as we have shown in this paper, should inform the development of potentially successful interventions against PRDs.

## Supporting information

S1 Text Questionnaire. (DOCX)

S1 Table CHECKLIST. (DOC)
