# Supplementary material for: Poverty and health among CDC plantation labourers in Cameroon: Perceptions, challenges and coping strategies
Source: PLoS Negl Trop Dis. 2017 Nov 20;11(11):e0006100. doi: 10.1371/journal.pntd.0006100 (PMC5714393; doi:10.1371/journal.pntd.0006100)
Supplement: S1 Text — (DOCX) [file pntd.0006100.s001.docx]

**Part 1: Background Information:**

1. Name:-_______________________________________________________________________
2. Age:____________________________________________________________________
3. Gender: Male Female
4. Marital status: Married single divorced widowed

separated

1. Educational level: No formal education FSLC GCE O’L GCE A’L

University technical/vocational training

1. Employment status: employed for wages house wife student self-employed retired
2. Income level (thousands): <20 between 20 and 50 between 50 and 100 > 100
3. Religion: Christian Muslim other______________

**Part 2: Housing Questions:**

1. Are you the owner of the house or is it a rented house?

Owner renting other arrangement--------------------

1. Was it easy for you to get a house to stay?

Yes No

1. Is your house affordable?

Yes No

1. How many people are living in your house?

1 2- 5 6-10

1. How many rooms do you have?

1 2 3 >3

1. Do you have enough space in your house for you (and your household)?

Yes no

1. Do you have enough privacy for yourself in the house?

Yes no

1. Have you made extensions/adjustments to increase space in your house?

Yes no

1. Is it easy to keep your house clean?

Yes no

1. Does the house protect you well against rain, heat/cold and wind?

Yes no

1. Does the house protect you well against mosquitos, rats or other vermin?

Yes no

1. Does your house protect you from thieves?

Yes no

1. Do you feel safe in your house during the night?

Yes no

1. Which parts of the house would you like to see improved?

Every part kitchen living rooms bedrooms

No part

**Part 3: Food questions**

1. How many meals do you eat daily?

1 2 3 4 >5

1. Is food readily available i.e. easy to get or buy for you?

Yes no sometimes

1. Is food easily affordable for you?

Yes no sometimes

1. Are there days in which you miss one or more meals?

Yes no sometimes

vvv

1. Why would you miss a meal? ______________________________________________________________________
2. Do you eat breakfast at the same hour each day?

Yes no

1. Do you eat lunch at the same hour each day?

Yes No

1. Do you eat dinner the same hour each day?

Yes No

1. Do you usually share your meal with others?

Yes no

1. How often do you take snacks between meals?

Never once twice, thrice >thrice

1. How much time do you spend on cooking food each day ?

0 minute 30minutes ………(continued on next page)

1 hour 2 hours 3 hours >3hours NA

1. How much time do you spend buying food at the restaurant each time?

30minutes 1 hour 2 hours 3 hours >3hours

NA

1. Where do you get the food you cook from?

Market farm garden other source______________________

1. Do you cook your meals?

Yes no

1. If yes, what do you use to cook your meals?

Fire side gas cooker charcoal pot saw dust.

1. Do you consider that your diet is balanced?

Yes No

1. If No, which elements are lacking usually?

Common grains/cereals/bread meat/fish/dairy vegetables fruits

1. Which of these do you eat daily?

Pulses tubers meat (beef, chicken fish) vegetables fruits

1. Do you take dairy or milk products every day?

Yes no

1. Is there someone who provides you with food when you are not well?

Yes no

1. Who provides you with food?

Mother father child relative neighbour

1. Are there specific food items you eat when you are not well?

Yes no

1. If yes, which are these ________________________________________________________________________
2. Are there some foods you do NOT eat when sick?

Yes no

1. Which are these?_____________________________________________________
2. Are there certain foods you eat for prevention of disease?

Yes no

1. If yes, which___________________________________________________________________
2. Do you eat or drink herbs, roots or other parts of plants to cure disease or illness.

Yes no

1. Which are these?_____________________________________________________

**Part 4: Water: Rapid assessment of drinking water quality (RADWQ):**

1. What is the main source of drinking water for members of your household?

Stream public tap well house taps

1. If not from house taps, how long does it take to go there, get water, and come back?

5mins 10mins 30mins >30mins

1. Who usually goes to this source to fetch the water for your household?

Mother father children relatives

1. Do you treat your water in any way to make it safer to drink even if carried from house taps?

Yes no

1. What do you usually do to the water to make it safer to drink?

Boil filter nothing Other____________

1. How do you store drinking water?

In a bucket in bottles in a pot other_____________

1. Are water cuts frequent in your neighbourhood?

Yes No

1. If yes, how do you get water in this situation/ how do you cope?

_____________________________________________________________

1. What other drinks do you usually take?

Tea coffee beer wine sweet drinks (sodas)

1. What kind of toilet facility do members of your household usually use?

Aqua privy toilet pit toilet water closet syst none

1. Do you share this facility with other households?

Yes no

1. If yes, how many households use this toilet facility?

1 2-5 5-10 >10

1. The last time the youngest child (less than 3 years) passed stools, what was done to dispose of the stools (camps)?

Dustbin toilet bush not applicable (NA)

1. Have you or any household member been sick from waterborne diseases in the last 6 months?

Yes no

1. Have you or any household member been sick from waterborne diseases in the last two weeks?

Yes no

**Health seeking behaviour using health belief model questionnaire**

1. Which of these are common in your area?

Malaria Diarrhoea HIV/AIDS Tuberculosis cholera STIs typhoid meningitis other_______________________

1. Why do you think this (these) is (are) common?

Poverty poor hygiene lack of knowledge poor education Climate

Other ________________

1. Which of these would you relate to poverty?

Malaria diarrhoea HIV/AIDS Tuberculosis cholera STIs typhoid other_______________

1. Which of these describes how you would normally respond to malarial attack?

Self-medication buy from road-vendor buy from big pharmacy buy from a small pharmacy go to hospital drink herbs go to a traditional doctor

1. Which of these describes how you would most likely seek health care if you were not well?

Formal (CDC hospital/ clinics) informal relational (family, friends or neighbours)

Informal personal (yourself, read med books or internet) other

1. What will determine that you seek formal or hospital healthcare?

Money(having) distance to healthcare centre duration of illness type and severity of disease attitude of hospital staff availability of drugs fear time

1. How soon would you seek formal or hospital health care?

Immediately after self-medication fails When illness is severe

When illness is long-lasting when pain is too much never other
